# Biochemical mechanisms of dose-dependent cytotoxicity and ROS-mediated apoptosis induced by lead sulfide/graphene oxide quantum dots for potential bioimaging applications

**DOI:** 10.1038/s41598-017-13396-y

**Published:** 2017-10-10

**Authors:** Mahdi Ayoubi, Parvaneh Naserzadeh, Mohammad Taghi Hashemi, Mohammad Reza Rostami, Elnaz Tamjid, Mohammad Mahdi Tavakoli, Abdolreza Simchi

**Affiliations:** 1 Department of Materials Science and Engineering, Sharif University of Technology, P.O. Box, 11365-11155 Tehran, Iran; 2 Department of Pharmacology and Toxicology, Faculty of Pharmacy, Shahid Beheshti University of Medical Sciences, P.O. Box, 14155-6153 Tehran, Iran; 3 Department of Nanobiotechnology, Faculty of Biological Sciences, Tarbiat Modares University, P.O. Box, 14115-175 Tehran, Iran; 4 Institute for Nanoscience and Nanotechnology, Sharif University of Technology, P.O. Box, 11365-11155 Tehran, Iran

## Abstract

Colloidal quantum dots (CQD) have attracted considerable attention for biomedical diagnosis and imaging as well as biochemical analysis and stem cell tracking. In this study, quasi core/shell lead sulfide/reduced graphene oxide CQD with near infrared emission (1100 nm) were prepared for potential bioimaging applications. The nanocrystals had an average diameter of ~4 nm, a hydrodynamic size of ~8 nm, and a high quantum efficiency of 28%. Toxicity assay of the hybrid CQD in the cultured human mononuclear blood cells does not show cytotoxicity up to 200 µg/ml. At high concentrations, damage to mitochondrial activity and mitochondrial membrane potential (MMP) due to the formation of uncontrollable amounts of intracellular oxygen radicals (ROS) was observed. Cell membrane and Lysosome damage or a transition in mitochondrial permeability were also noticed. Understanding of cell-nanoparticle interaction at the molecular level is useful for the development of new fluorophores for biomedical imaging.

## Introduction

Quantum dots (QD) are semiconductor and light-emitting nanoparticles with unique chemical, electrical and optical properties. Due to the quantum confinement effect^1^, QD have size-tunable light emission and broad absorption spectra. These characteristics along with their resistance to photoetching allow bright and concurrent excitation of multiple fluorescence colors, offering superb advantages over organic dyes and fluorescent proteins^2^. The unique optical properties of QD and their least absorption by body tissue have made them suitable for dynamic imaging at the single-molecule level^3^ and ultrasensitive biomedical diagnostics^4^. In spite of these advantages, the presence of heavy metal ions and the potential toxicity of QD, their chemical instability in aqueous solution (due to reactive dangling bonds on their surfaces), and blinking (rapid on-and off light emission) remain major concerns^5–7^. In order to attain water-soluble, non-aggregated and photo-stable QD, it is inevitable to modify their surface chemistry by hydrophilic polymers^8^. The polymer coating also reduces the propensity of nonspecific binding to proteins and cellular membranes^9^. Nevertheless, polymer-coated QD often suffer from reduced quantum efficiency, a large hydrodynamic size, and chemical heterogeneity^10^. A considerable interest still remains for developing functional and bright-emitting QD with optimized hydrodynamic size. Moreover, the inorganic fluorophore must resist nonspecific interactions and site-specific ligand conjugation^11^.

Lead sulfide (PbS) nanoparticles are particularly attractive for optoelectronic and biomedical applications. The absorption spectra of PbS QD can span from low to mid infrared range (dependent on their size) with a large Bohr radius (18 nm) and long fluorescence lifetime (up to microseconds)^12^. The photoluminescence quantum yield (PLQY) remains high even after the transfer to water^13^. The high atomic number of lead also provide good X-ray absorption under clinical voltages, which makes them suitable as contrast agent for X-ray and CT imaging^14^. However, PbS QD are toxic due to unwanted leakage of lead ions in biological environment^15^. Additionally, water solubility and stability of these nanocrystals are major concerns because the nanocrystals are mainly synthesized by organic chemistry. Chemical synthesis of QD in organic solvents often yields monodispersed particles with well-passivated surface functional groups (because lead oxides are easily form on the dangling bonds with PLQY loss)^16^. Therefore, transferring of the hydrophobic QD to water while keeping the high quantum efficiency is tricky. Hinds *et al*.^17^ functionalized the mercaptodecan chain with tetraethylene glycol to replace the surface ligands of PbS QD to obtain water-soluble nanocrystals. The dots were stable in 4-(2-hydroxyethyl)-1-piperazineethanesulfonic acid (HEPES) buffer for 5 days and then settled down. Lin *et al*.^18^ transferred PbS QD to water through a ligand exchange process with polyacrylic acid. The nanocrystals showed a decreased PLQY (from 82% to 24%) after ligand exchange and complete loss of emission after few days. Wang *et al*.^19^ utilized green chemistry routes to prepare water-soluble PbS QD with a double coating of silica-polymer (PEG) layer. Yang *et al*.^20^ synthesized PbS-Bovine hemoglobin nanocubes using a protein based method. Deng *et al*.^21^ synthesized PbS QD with strong infrared fluorescence directly in aqueous solution using a lipoic acid as a stabilizer. The dots were highly stable in water with a lower toxicity than CdTe, CdHgTd, and HgTd quantum dots. Liang *et al*.^22^ used graphene quantum dots as an agent to transfer nanoparticles from non-polar to polar solvents. By photoluminescence study, energy transfer from graphene dots to nanoparticles was shown.

In spite of great potential of utilizing PbS QD for biomedical applications, their toxicity remains a major concern, which inhibits their usage for diagnosing and treating patients. The toxicity of PbS QD can be ascribed to different sources, but like other nanoparticles leakage of toxic metal ions, imbalance in systemic manifestation of reactive oxygen species (ROS) and detoxification the reactive intermediates induced by intracellular oxidative stresses, and autophagy have major roles^23^. It is well known that lead is hazardous to a number of body functions such as the central nervous, hematopoietic, hepatic and renal system. ROS are chemically reactive chemical species that have imperative roles in cell signaling and homeostasis^23^. A dramatic increase in ROS level, for example through the inhibition of mitochondrial electron transfer chain (ETC) complexes, may result in important damage to cell structures and functions. Many studies have shown that mitochondrial-produced ROS assists the mitochondrial permeability transition (MPT) which is the key important motivations for apoptosis and necrosis^24^. Autophagy is a lysosome-dependent cellular degradation process, a major mechanism of cell death^24^. There is a myriad of scientific literatures and review papers on the cytotoxicity evaluation of nanomaterials, particularly nanoparticles and quantum dots, and their effects on the biological systems. Major toxicity mechanisms include contamination with toxic elements and generation of radical species, high surface charge and fibrous structure, morphological issues (e.g. needle-like or sharp-edged particles), and modified physicochemical properties due to very high surface to volume ratio and enhanced reactivity^25–27^. It is generally agreed that the cytotoxicity of QD depends on their properties (composition, size, shape, aggregation, surface charge, redox activity, surface coating, degree of stability, etc), the responding cell type, routes of exposure, processing parameters and environmental conditions^28–32^. Therefore, results on cell response to QD are often inconsistent and contradictory. The liberation of transition or heavy metal ions (e.g. Pb^2+^ and Cd^2+^) from QD intact with cells induces toxic effects through conventional mechanisms of metal toxicity such as damage to lipids, proteins, enzymes and DNA via the production of free radicals or replacing other cations which disrupt biological metabolism of the cells^33–35^. Free radical intermediates or redox active groups present on the surface of QD can also induce oxidative stress leading to ROS-medicated apoptosis^36,37^. Therefore, many studies have been performed to modify the surface of QD in order to enhance their chemical stability and to tailor their physicochemical properties.

Surface functionalization of PbS QD to avoid lead ion leakage and prevent ROS production irregularities and autophagy is vital if the nanocrystals are to be used for biomedical applications. In contrast to previous investigations, which surface functionalization has been performed by hydrophilic polymers^10^, we present a chemical strategy to prepare quasi core-shell PbS/rGO quantum dots. It is demonstrated that the heterostructure nanocrystals have very high solubility/dispersibility in water (concentration as high as 50 mg/ml), long stability (up to months), and a high PLQY (28%). The dose-dependent toxicity of the nanoparticles in cultured human mononuclear cell is shown. Molecular mechanisms of interaction with the cells are also explained.

## Methods

### Materials

To prepare PbS QD, lead (II) oxide powder (PbO, 99%), oleic acid (OA, technical grade 90%) and anhydrous solvents including toluene, octane, methanol and isopropanol were purchased from Merck & Co. (Darmstadt, Germany). Bis(trimethylsilyl) sulfide (TMS, Strem, 97% purity) and 1-octadecene (ODE, Sigma-Aldrich, technical grade 90%) were utilized without purification. A high purity graphite rod and utilized for the preparation of graphene nanosheets.

We used MTT (3-[4,5-dimethylthiazol-2-yl]-2,5-diphenyltetrazolium bromide) to assay activity of mitochondrial complex II (succinate dehydrogenase). The formazan crystals were dissolved by dimethyl sulfide (DMSO). We evaluated ROS generation and MMP by 2′,7′-dichlorofluorescein diacetate (DCFH-DA) and Rhodamine 123 (Rh 123) probes. For the evaluation of lipid peroxidation, Malondialdehyde (MDA), Thiobarbutiric acid (TBA), n-butanol, and Tetramethoxypropane (TEP) were utilized. GSH and GSSG were determined by OPA and NEM probes. Lysosomal membrane integrity was analyzed by acridine orange. We used different buffers including Tris-HCl, sucrose, MgCl_2_, KCl, MnCl_2_, potassium phosphate 2-aminoethylether- N, N, N′, N′-tetraacetic acid (EGTA), Ethylene ediamine tetra acetic acid (EDTA) and Na_2_HPO4. All chemicals were supplied by Sigma Chemical Co. (St. Louis, MO, USA) and were of analytical grade, high performance liquid chromatography (HPLC) grade or the best pharmaceutical grade. All materials were dissolved in saline.

### Synthesis of quasi core-shell nanocrystals

To synthesis the heterostructure fluorophore, ultrafine graphene oxide nanosheets were synthesized by electrolysis of the graphite rod in an aqueous solution of NaOH (0.1 M). A glass vessel (100 mL) containing a working electrode (graphite) as anode and a counter electrode (platinum foil with thickness of 1 mm) as cathode were utilized. The distance between the parallel electrodes was 1 cm. A constant current of 120 mA/cm^2^ were applied for 30 min. Afterwards, Na^+^ and OH^−^ ions were separated by dialysis tubing 3.5 kDa; then, the solution was dried at 373 K and dispersed in 1MP (1 mg/ml) (1-Methyl-2-pyrrolidone). In another flask, 0.7 g of PbO were mixed with 28 mL of 1MP and then 10 ml of the graphene oxide suspension (1 mg/ml) was added to the flask maintained at 110 °C and stirred for 14 h under argon stream. Afterwards, a solution containing 210 μl of TMS dissolved in 10 mL of 1MP was swiftly injected into the flask and vigorously stirred for 2 h. The reaction product was separated by centrifuge (SIGMA, Germany) at 20000 rpm for 20 min and the remained solution was separated as product. Ions were separated from the solution by dialysis tubing 3.5 kDa. The hybrid nanocrystals were then precipitated by vacuum evaporation and finally dispersed in PBS.

### Materials characterizations

The size and morphology of the nanocrystals were studied by a high-resolution transmission electron microscope (HRTEM, JOL, JEM-2100, Japan) equipped with an energy-dispersive X-ray spectroscopy (EDS). Phase characterizations were performed by employing an X-ray diffractometer (Stone Sandi P, USA) utilizing a Cu Kα radiation (1.54 Angstrom). The absorption spectrum of the nanocrystals was recorded on a UV-Vis-NIR Carry 500 spectrometer (Varian, USA). Fourier transformed infrared spectroscopy (FTIR) was performed by a Perkin-Elmer instrument (RX, USA). Raman shifts were measured by a InVia (Renishaw AB, Sweden) spectrometer using an incident laser light with 514.5 nm wavelength. Surface analysis was performed by X-ray photoelectron spectroscopy (XPS). A hemispherical analyzer with an Al Kα X-ray source (1486.6 eV) at 10^−7^ Pa was employed. Deconvolution of XPS peaks was performed on Gaussian components after a Shirley background subtraction. The photoluminescence (PL) emission spectrum of the nanocrystals was recorded on a FLS920P Edinburgh instrument (Kirkton Campus, UK) equipped with a cryogenically cooled photomultiplier (R5509-43, Hamamatsu). For steady-state spectra, a 450 W continuous xenon arc lamp was utilized. For lifetime measurements (PL decay) a picosecond pulsed diode laser (EPL-405, excitation wavelength 405 nm, pulse width: 49 ps) was used. A stretched exponential function with two characteristic parameters of τ (decay time) and β (stretch parameter) was utilized for the curve fitting. To determine the photoluminescence quantum yield (PLQY) of the nanoparticles, an absolute method^38^ was used. The fluorescence spectrometer equipped with an integrating sphere with BENFLECR coated inner face (Edinburgh Instruments) was employed.

### *In vitro* toxicology of heterostructure quantum dots

Effects of nanoparticles on the mononuclear human cells were examined by various biological assays. Fresh, healthy and untreated bloods of 15 donors were utilized for these studies. Peripheral blood (PB) samples were obtained after informed consent on age-matched controls (20 to 25 years), and health assurance was confirmed by clinical examination as well as morphological and immunological criteria. The study was approved by the research ethics committee of the Shahid Beheshti University of Medical Sciences (Tehran, Iran) and all the patients signed an informed consent form. Instruments utilized for the *in vitro* toxicology investigations include a MCO 17A1 CO_2_ incubator (Sensor Sanyo IR, Japan), vapor bath stark eliwellewpc 800 (UK), Harrier 18/80 refrigerated centrifugation (Sanyo, Japan), UV/Visible spectrometer (Shimadzu 160 ABB, Japan), floremetry (Shimadzu RF-5000, Japan), digital balance (Shimadzu 20 E8 330 H, Japan), shaker (REAX2000, Iran), ELISA reader (In finite 200 M, TECAN, Rainbow Thermo, Austria), and BD Biosciences FACS Calibure TM flow cytometer equipped with a 488 nm argon ion laser and a 530 nm band pass filter (FL-1 channel). Details of experimental analyses are explained below.

### Isolation of human Lymphocytes cells

Human blood was obtained from normal donors by vein puncture and was mixed undiluted with heparin at 10 U/mi. This was layered on lymphocyte separation medium and centrifuged at 400 × g for 25 roll. The mononuclear cells (MNCs) were collected from the interface and washed three times at 440 × g for 10 min with RPMI1640 medium. Fresh plastic tubes were used at each centrifugation step. The viability of the cells was always > 99.5% as estimated by trypan blue dye exclusion. Then, the mononuclear cells were separated and purified. Stock isotonic Percoll (SIP) was prepared by using nine parts of Percoll, 0.9 parts of 10 × HBSS and one part of 1 M MOPS (pH = 7.4). It was then diluted with HBSS + 10 mM MOPS (HBSSMOPS) medium to prepare different densities (1.04–1.08 g/~ at 21 °C) according to the regression equation described by Ulmer and Flad^39^.

### Cell viability assay and determination of critical toxicity concentration

Cell viability was assessed by 3-(4,5-dimethylthiazol-2-yl)-2,5- Diphenyl tetra zoliumbromide (MTT) staining^40^. Lymphocytes cells (1 × 10^4^ cells/well) were incubated in 96-well plates in the presence or absence of the nanoparticles for 6 h in a final volume of 50 ml. The absorbance was measured at 570 nm on ELISA reader.

### Reactive oxygen species and mitochondrial membrane permeability

Lymphocytes cells (1 × 10^6^ cells) were treated with the hybrid quantum dots for 6 and 12 h. After treatment, Lymphocytes cells were washed with PBS. H2DCFD and Rhodamin 123 (10 mM) were used to measure intracellular reactive oxygen species (ROS) and disrupted cell membrane. These agents diffuse into the cells causing de-esterification. Subsequent reactions with peroxides generate fluorescent 5-chloromethyl-2′, 7′ dichlorofluorescein (DCF). Mitochondrial membrane permeability (MMP) was determined by flow cytometry using a lipophilic cationic dependent fluorescent dyerhodamine (Rh123). Cells were read on the flow cytometer and light scattering were analyzed for at least 10000 counts per sample. A flow cytometer with the Flowing software-2-5-1, equipped with a 488 nm argon ion laser was used and fluorescence signals were obtained using a 530 nm band pass filter (FL-1 channel).

### Lipid peroxidation

The content of MDA was determined using the method of Zhang *et al*.^41^. The human Lymphocytes cells (1 × 10^6^ cells/well) were incubated with various concentrations of the nanoparticles for 1 h at 30 °C. Then, 0.25 ml sulfuric acid (0.05 M) was added to 0.2 mL cell fractions. Afterwards, 0.3 ml of a solution containing 0.2% TBA was added. All the microtubes were placed in a boiling water bath for 30 min. At the end, the tubes were shifted to an ice-bath and 0.4 mL n-butanol was added to each tube. The tubes were centrifuged at 3500 × g for 10 min. The amount of MDA formed in each of the samples was assessed through measuring the absorbance of the supernatant at 532 nm with an ELISA reader (Tecan, Rainbow Thermo, Austria). Tetramethoxypropane (TEP) was used as standard and MDA content was expressed as nmol/mg protein^41^.

### Glutathione disulfide and oxidized glutathione content

The ratio of reduced glutathione disulfide (GSH) to oxidized glutathione (GSSG) is a sensitive indicator of oxidative stress in cells^42^. Therefore, intracellular GSH and GSSG were determined based on spectrofluorometric method by employing O-Phthalaldehyde (OPA) and N-Ethylmaleimide (NEM) probe. Aliquots of the cell suspension (0.5 ml) that were previously stained with OPA and NEM probe (5 µM) were separated from the incubation medium by 1 min centrifugation at 1000 rpm. The cell pellet was then suspended in 2 ml of fresh incubation medium. This washing process was carried out twice to remove the fluorescent dye from the media. Each sample was measured in quarts cuvettes using a Shimadzu RF5000U fluorescence spectrophotometer set for at 495 nm excitation and 530 nm emission wavelengths.

### Lysosomal membrane integrity assay

Lymphocytes lysosomal membrane stability was determined from the redistribution of acridine orange as a fluorescent dye. Aliquots of the cell suspension (0.5 ml) that were previously stained with acridine orange (5 µM) were separated from the incubation medium by 1 min centrifugation at 1000 rpm. The cell pellet was then suspended in 2 ml of fresh incubation medium. This washing process was carried out twice to remove the fluorescent dye from the media. Acridine orange redistribution in the cell suspension was then measured fluorimetrically by the spectrophotometer set at 495 nm excitation and 530 nm emission wavelengths.

### Adenosine triphosphate synthase

The concentration of adenosine triphosphate (ATP) in mitochondria was determined by a bioluminescent somatic cell assay kit (sigma Aldrich.MO 63103, USA). In this measurement, it is assumed that the ATP content per viable cell remains fairly constant. The bioluminescence intensity was measured by a Sirius tube luminometer (Berthold Detection System, Germany).

### Statistical analysis

Results are presented as mean ± SD. All statistical analyses were performed using the SPSS software, version 20. Assays were performed 5 times and the mean was used for statistical analysis. Statistical significance was determined using the one-way ANOVA test, followed by the post hoc Tukey test. In some experiments, the two-way ANOVA test followed by the post hoc Bonferroni test was performed. Statistical significance was set at *P* < 0.05.

### Compliance of ethical standards

All procedures performed in this study were in accordance with relevant guidelines and regulations of the Sharif University of Technology and Shahid Beheshti University of Medical Sciences (Tehran, Iran). This study has been approved by the ethical committee and all the patients signed an informed consent form.

## Results

### Characteristics of heterostructure nanocrystals

**Figure 1:**
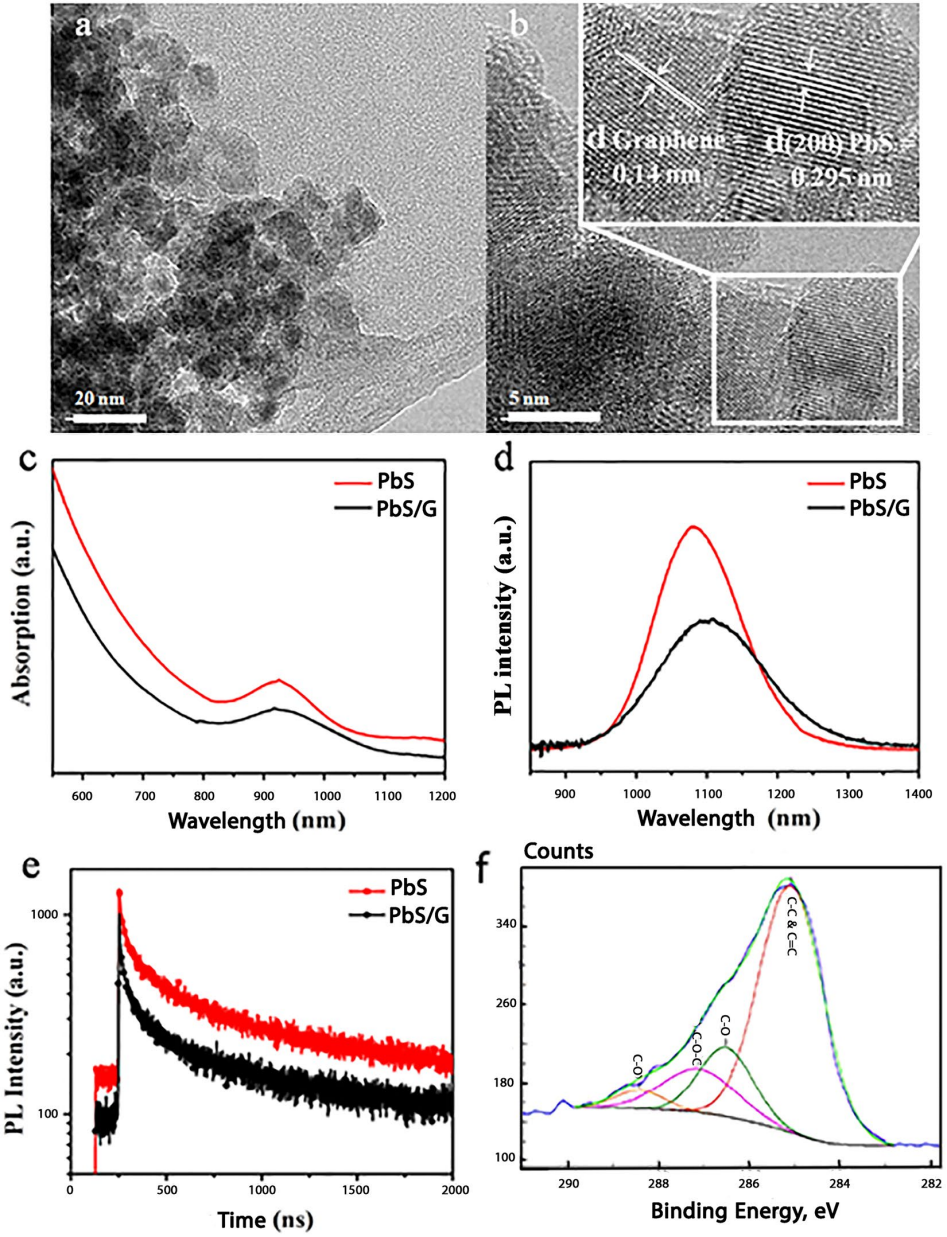
Characteristics of heterostructure semiconductor nanocrystals: (**a**,**b**) TEM images of the quantum dots. (**c**) UV-Vis spectra show a relatively broad absorption peak at around 940 nm. (**d**) Steady-state and (**e**) TRPL measurements indicate NIR emission at around 1100 nm with relatively long carriers’ lifetime. (**f**) Deconvoluted XPS C1s spectra reveal surface functional groups of the prepared QD.

Graphene oxide nanosheets were prepared by the electrolysis of graphite rod. As shown in Supplementary Electronic Information (ESI) S1, the graphene dots have uniform lateral dimensions of about 10 nm with a thickness of ~1 nm. The dots were injected into the hot batch of lead oxide to prepare the heterostructure nanocrystals. TEM study indicated that ultrafine particles with an average size of ~4 nm with a narrow size distribution were formed (Fig. 1a). From the high-resolution TEM study (Fig. 1b), it appeared that an ultrathin layer of graphene with hexagonal structure and an atomic distance of 0.14 nm was wrapped around the core particles. XRD study (ESI S2a) showed the presence of strong characteristic peaks of cubic lead sulfide (JCPDS 02-0699) with a weak characteristic peak of graphene at 26° (d-spacing of 0.34 nm).

Spectroscopic analyses were performed to study the absorption and emission response of PbS QD before and after processing with GO nanosheets. Figure 1c shows that both nanocrystals have a relatively broad and diffused absorption peak at around 940 nm. As compared with the pristine PbS QD prepared by the same method, a slight shift in the excitonic peak to higher wavenumbers with a reduced intensity is noticeable. PL spectra of the hybrid nanoparticles in steady-state and time-resolved conditions are shown in Fig. 1d and e, respectively. PbS QD show a bright emission at near infrared region (around 1100 nm) with a high intensity. The heterostructure nanocrystals exhibit broader PL peak with lower intensity (due to fast extraction of carriers) and slightly red-shift. However, no change in PLQY after processing with graphene nanosheets is seen; both materials have ~28% quantum efficiency, as reported elsewhere^43^. On the other hand, time-resolved PL measurement (TRPL) indicates faster charge transfer (0.9 µs) for the hybrid quantum dots as compared with the pristine PbS QD (1.1 µs) layer. It should be mentioned that this experiment was performed on a thin TiO_2_ layer (300 nm) as the electron transfer layer. Fitting parameter of TRPL curves are shown in ESI Table S1. From FTIR analysis (see ESI S2b), it was noticed that upon synthesis of PbS QD, some of the functional groups of graphene sheets were reduced. To support this finding, XPS was employed (ESI S2c). Deconvoluted XPS C1s spectra (Fig. 1f) indicates that the heterostructure semiconductor particles have many surface functional groups^44^. The presence of carboxylate and epoxy groups on the surface of hybrid nanocrystals possesses a good water dispersibility and stability, which are essential for biomedical applications. Raman spectrum of the heterostructure nanocrystals is shown in ESI S2d. The main characteristic peaks of graphitic materials, i.e. G and D bands, were detectable. The noisy bands are most probably originated from the strain induced symmetry breaking, due to the bending of the graphene layer on the surface of PbS QDs^45^. The peak at 602 cm^−1^ are from 3LO phonon modes (longitudinal optical phonons) while the weak peak at 966 cm^−1^ may be related to the photodegradation of PbS.

### Concentration dependent cell viability studies

**Figure 2:**
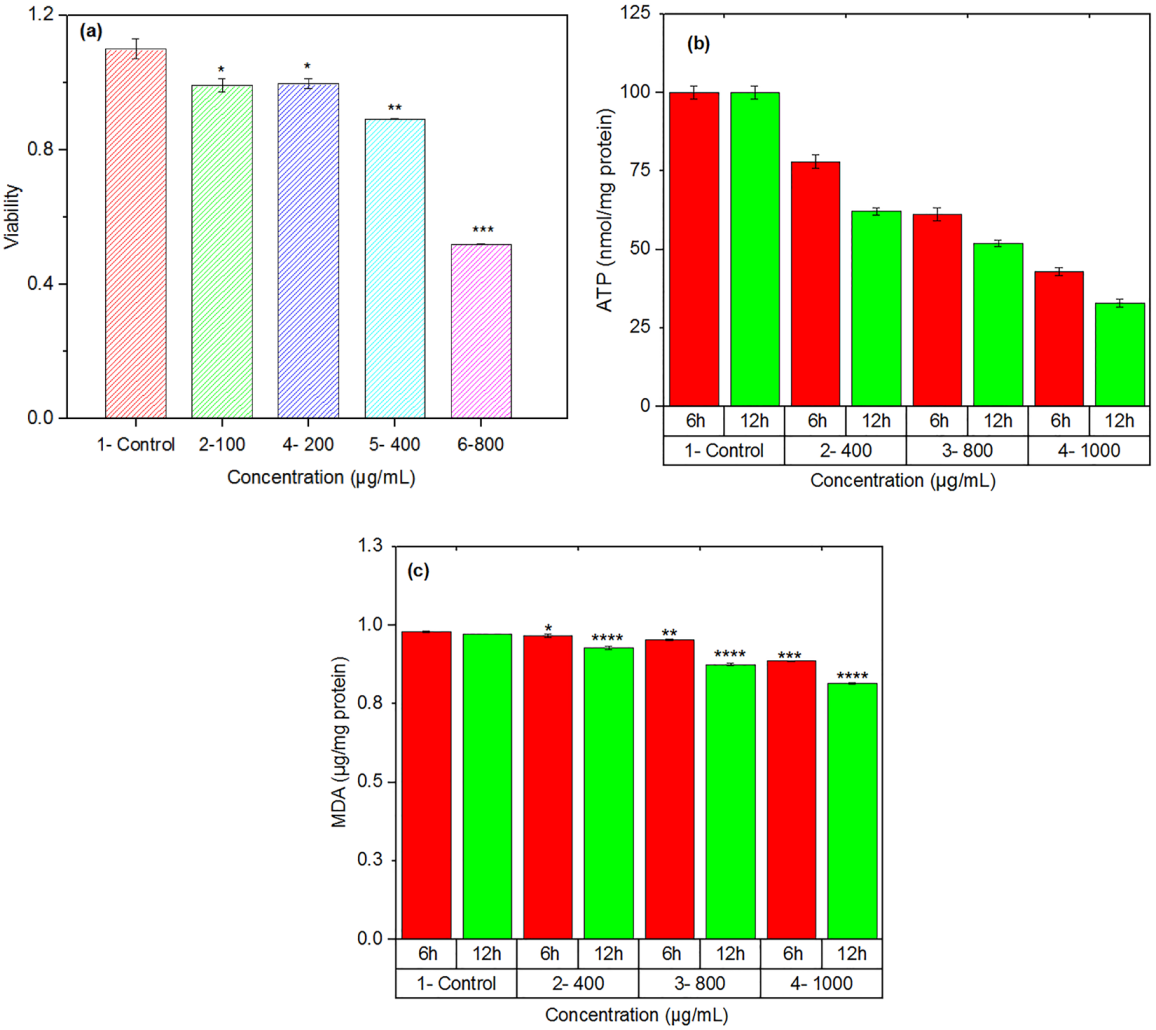
(**a**) The effect of hybrid nanoparticles on the cell viability (SDH activity). (**b**) Changes in the ATP production shows the effect of nanoparticles on the mitochondrial respiration. (**c**) The nanoparticles induced lipid peroxidation in isolated mononuclear cells. Data represented as mean ± SD of data determined from three separate experiments. Values represented as mean ± SD (n = 3). *P < 0.05; **P < 0.01; ***P < 0.001 and ****P < 0.0001; compared with control cell.

For the measurement of cell viability, we assessed succinate dehydrogenase or SDH activity using the MTT test after 6 h incubation of Lymphocytes cells with the heterostructure nanoparticles at different concentrations ranging from 10 to 800 µg/ml. It was found that the nanoparticles did not show a significant toxicity up to concentration of 200 µg/ml (Fig. 2a). At higher concentrations, however, the mitochondrial metabolic conversion of MTT to formazan was decreased, showing toxicity of the hybrid nanoparticles.

### Effect of heterostructure nanoparticles on mitochondrial reactive oxygen species

**Figure 3:**
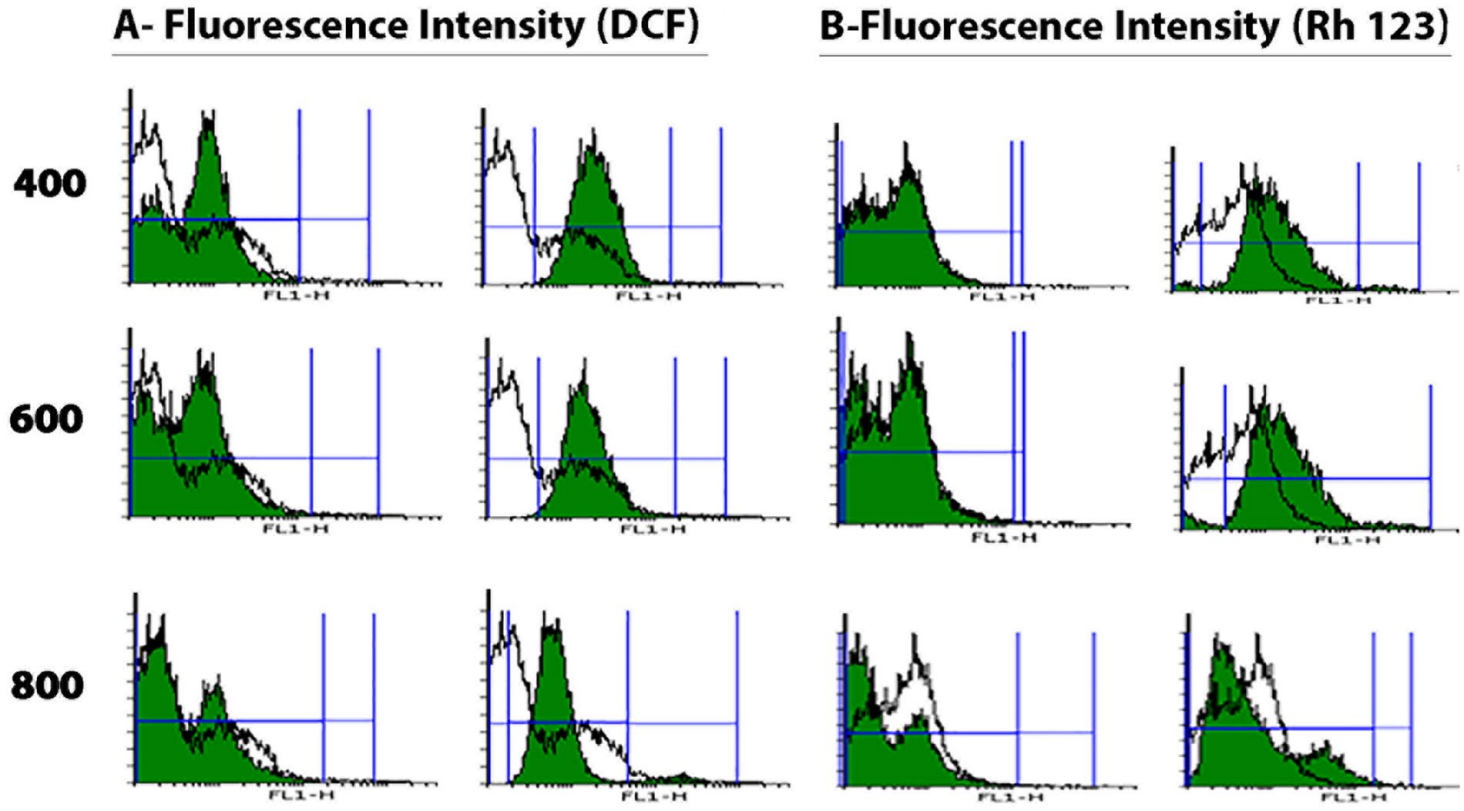
Flow cytometry graphs showing the effect of heterostructure quantum dots on (**a**) ROS formation and (**b**) MMP of lymphocytes cells.

To understand possible toxicity mechanism at the high concentration, the effect of nanoparticles on the formation of ROS was assayed. The flow cytometric graphs are shown in Fig. 3a. The significant peak shifting indicates that a high amount of ROS are formed in the lymphocytes cells exposed to the nanoparticles. It is pertinent to point out that chronic increase in the ROS production induces ROS-associated damages in DNA, proteins, and lipids, which finally cause cell dysfunctions^46^. Since mitochondria are the main cause of ROS in the cell, there should be a relationship between mitochondrial membrane potential (MMP) and the rate of ROS formation. Therefore, we studied possible influence of the heterostructure nanoparticles on MMP. From flow cytometric graphs (Fig. 3b), it was concluded that a high concentration of heterostructure QD could decline MMP of lymphocytes cells. Thus, it suggested that the superoxide anion, as undesired by-product of mitochondrial oxidative phosphorylation, was triggered by a leak of electrons from the mitochondrial respiratory chain. Since mitochondrial electron transfer chain is required for ATP production and the nanoparticles impairs the mitochondrial respiration, we measured the ATP level in isolated mitochondria from lymphocytes cells following the addition of nanoparticles. As shown in Fig. 2b, the nanoparticles significantly decreased the ATP content in a concentration dependent manner. The ATP reduction indicates the cell metabolism dysfunction.

### Lipid peroxidation, thiols metabolism and redistribution of lysosome

**Figure 4:**
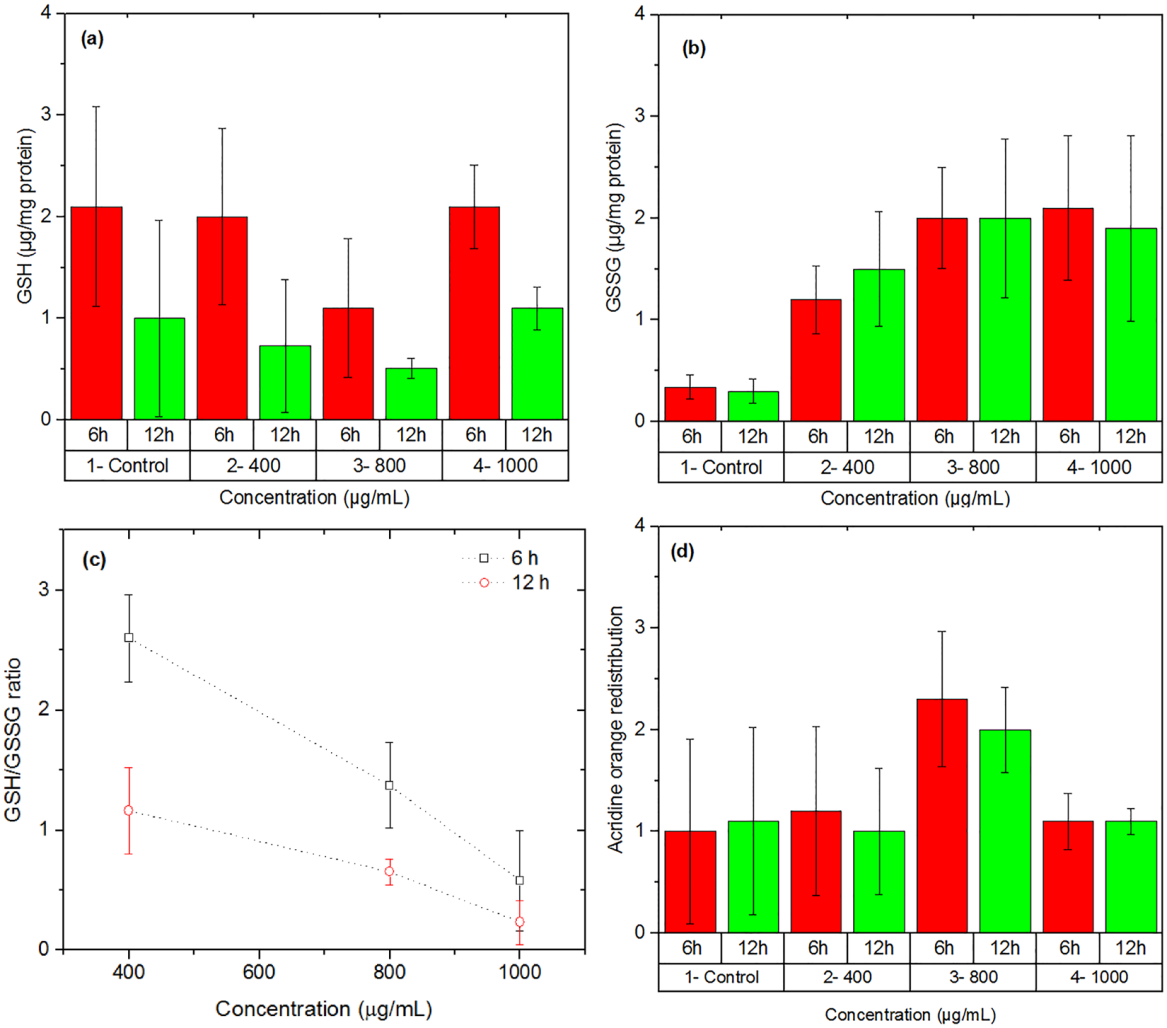
Effect of hybrid nanoparticles on glutathione in both (**a**) reduced (GSH) and (**b**) oxidized (GSSG) states. (**c**) The ratio of GSH/GSSG indicates the role of nanoparticles on the oxidative stress. The dot lines only show the trend of variations to help eyes. (**d**) Changes in acridin orange (a lysosomotropic agent) indicate possible lysosomal damage by the nanoparticles.

Experiments showed a high rate of ROS formation upon exposure of a concentrated (≥400 µg/ml) nanoparticles to the human cells. ROS combine with a hydrogen atom to make water and a fatty acid radical; hence, through the chain reaction mechanism oxidative degradation of lipids occurs that can lead to cell membrane damage^47^. We examined the concentration dependent formation of MDA production for the hybrid nanoparticles. As Fig. 2c shows, the nanoparticles induce lipid peroxidation at high concentration. Since thiol metabolism has special relevance to understanding the cell’s defense against toxicant exposure, the concentration of glutathione in cells in both reduced (GSH) and oxidized (GSSG) states was determined after incubation with the hybrid nanoparticles using OPA as probe. Figure 4a,b show the results. The ratio of GSH to GSSG may be used as a marker of oxidative stress because GSH is considered to be one of the most important scavengers of ROS. Our results indicate that the GSH/GSSG ratio decreased with increasing the concentration of nanoparticles (Fig. 4c). Possible effect of hybrid nanoparticles on lysosomal damage was also studied by acridin orange (a lysosomotropic agent) as probe. As Fig. 4d shows, the nanoparticles caused significant damage to lysosomal membrane at concentrations ≥ 800 µg/ml. This damage can cause caspase dependent apoptosis or even necrosis due to high level of lysosomal membrane permeabilization.

## Discussion

Hybrid quantum dots based on quasi core-shell lead sulfide-graphene as a biocompatible near infrared probe with potential application in bioimaging were introduced. To prepare the quantum dots, graphene oxide nanosheets were utilized instead of organic ligands such as oleic acid (OA) to passive the deep trap states. The ligand also controls the nucleation and growth of nanocrystals to a desirable degree in order to prevent the bulk material to form. When lead oxide is mixed with the graphene dots in 1MP, the lead ions are prone to chemisorb on the functional groups of the graphitic materials^45^. Introducing the highly active sulfur precursor (TMS) leads to heterogeneous nucleation of lead sulfide, which are then grown by further absorption of lead ions from the solvent. Due to the high mobility of free ions, they can easily reach the nucleus to react with sulfur to form lead sulfide on the surface of the growing nanocrystals. The presence of graphene dots prevent excess growth of the lead sulfide nanocrystals and encapsulate the core. Because of geometrical limitations, this encasement is not completely executed and quasi core/shell quantum dots are attained. Peak spliting in the Raman spectrum of the hybrid quantum dots determines bending of the small nanosheets around the core, while XPS study indicates partial reduction of the surface functional groups of the graphene oxide during processing.

The critical concentration, which the hybrid quantum dots are not toxic to human mononuclear blood cells, was determined by succinate dehydrogenase or SDH activity using the MTT test. It was shown that up to 200 µg/ml the hybrid nanoparticles are safe. At higher concentrations, a decrease in the mitochondrial metabolic conversion of MTT to formazan following was noticed. Our study determined that a reduction in complex II activity ameliorates mitochondrial respiration rates occurred. A rapid increase in ROS formation along with the decreased GSH/GSSG ratio (related to the thiol metabolism) was also noticed. This finding indicated oxidative stress as the main toxicity mechanism. It is known that GSH is a required component for preservation of thiol groups which protects mitochondria against permeability transition or opening of MPT pores and oxidative stress. The inverse linear relationship between the ROS level and the GSH level indicated that free radical species were generated by exposure to the hybrid quantum dots with decreased mitochondrial and cellular antioxidant levels. On the other hand, measurement of lipid peroxidation indicated a significant increase in the MDA level. Therefore, oxidation of lipid membranes resulted in disruption of mETC, and consequently collapsing of MMP^48^. This phenomena could cause cytochrome c explosion from mitochondria to the cytosol leading to the apoptosis signaling^49^. Additionally, the results of ATP measurement showed that the superoxide anion was triggered by a leak of electrons from the mitochondrial respiratory chain. Therefore, the hybrid nanoparticles at high concentrations impaired the mitochondrial respiration. The toxic effect of quantum dots on human lymphocytes cells could be due to their direct influence on enzymatic activity such as inhibition of mitochondrial ATPase. The decreased ATP level along with damaged lysosome could orchestrate apoptosis signaling in human lymphocytes cells^50^.

## Conclusions

In summary, quasi core/shell lead sulfide/reduced graphene oxide nanoparticles were synthesized by a modified hot injection process. The quantum dots with an average diameter of about 4 nm compose of cubic PbS core and a thin layer of graphene shell. Spectroscopic analyses indicate that the quantum dots are photoactive with bright light emitting in near infrared region (1100 nm) with relatively long carriers’ lifetime (about 1 µs). The quantum dots are dispersible in aqueous solutions while the graphitic shell passivate their dangling bonds and deep trap states. To study the potential application of the hybrid quantum dots for bioimaging, their cytotoxicity intact with human mononuclear cells were studied *in vitro*. The light-emitting quantum dots did not show major cytotoxicity at concentrations ≤ 200 µg/ml. The precise mechanisms underlying the toxicity of the heterostructure quantum dots at higher concentrations was studied. It was shown that oxidative stress in human mononuclear cells was directly involved. The reduced cell viability was associated with significant increase in intracellular ROS level and toxic alterations in mitochondria and lysosomes. These effects triggered depleted glutathione and lipid peroxidation. It was also shown that the oxidative stress damaged mitochondrial membrane causing cytochrome c expulsion along with decreased ATP level, which ultimately led to cell death.

## Electronic supplementary material

Electronic Supplementary Information

## References

[1] Wise FW (2000). Lead salt quantum dots: the limit of strong quantum confinement. Accounts of Chemical Research.

[2] Wang F, Tan WB, Zhang Y, Fan X, Wang M (2005). Luminescent nanomaterials for biological labelling. Nanotechnology.

[3] Michalet X (2005). Quantum dots for live cells, *in vivo* imaging, and diagnostics. Science.

[4] Gao X, Chan WC, Nie S (2002). Quantum-dot nanocrystals for ultrasensitive biological labeling and multicolor optical encoding. Journal of biomedical optics.

[5] Resch-Genger U, Grabolle M, Cavaliere-Jaricot S, Nitschke R, Nann T (2008). Quantum dots versus organic dyes as fluorescent labels. Nature methods.

[6] Ma J (2007). Photochemical instability of thiol-capped CdTe quantum dots in aqueous solution and living cells: process and mechanism. Journal of Physical Chemistry B.

[7] Mahler B (2008). Towards non-blinking colloidal quantum dots. Nature materials.

[8] Yildiz I, McCaughan B, Cruickshank SF, Callan JF, Raymo FM (2009). Biocompatible CdSe− ZnS core− shell quantum dots coated with hydrophilic polythiols. Langmuir.

[9] Mozafari M, Moztarzadeh F (2010). Controllable synthesis, characterization and optical properties of colloidal PbS/gelatin core–shell nanocrystals. Journal of colloid and interface science.

[10] Tomczak N, Liu R, Vancso JG (2013). Polymer-coated quantum dots. Nanoscale.

[11] Muro E (2010). Small and stable sulfobetaine zwitterionic quantum dots for functional live-cell imaging. Journal of the American Chemical Society.

[12] Cassette E (2013). Design of new quantum dot materials for deep tissue infrared imaging. Advanced drug delivery reviews.

[13] Zhao H, Wang D, Zhang T, Chaker M, Ma D (2010). Two-step synthesis of high-quality water-soluble near-infrared emitting quantum dots via amphiphilic polymers. Chemical Communications.

[14] Yu S-B, Watson AD (1999). Metal-based X-ray contrast media. Chemical reviews.

[15] Novelli AA, Losso C, Ghetti PF, Ghirardini AV (2003). Toxicity of heavy metals using sperm cell and embryo toxicity bioassays with Paracentrotus lividus (Echinodermata: Echinoidea): comparisons with exposure concentrations in the lagoon of Venice, Italy. Environmental Toxicology and Chemistry.

[16] Pahari SK, Adschiri T, Panda AB (2011). Synthesis of monodispersed nanocrystalline materials in supercritical ethanol: a generalized approach. Journal of Materials Chemistry.

[17] Hinds S (2007). NIR-emitting colloidal quantum dots having 26% luminescence quantum yield in buffer solution. Journal of the American Chemical Society.

[18] Lin W (2008). Highly luminescent lead sulfide nanocrystals in organic solvents and water through ligand exchange with poly (acrylic acid). Langmuir.

[19] Wang D (2012). ‘Green’-synthesized near-infrared PbS quantum dots with silica–PEG dual-layer coating: ultrastable and biocompatible optical probes for *in vivo* animal imaging. Nanotechnology.

[20] Yang G, Qin D, Zhang L (2014). Controllable synthesis of protein-conjugated lead sulfide nanocubes by using bovine hemoglobin as a capping agent. Journal of nanoparticle research.

[21] Deng D (2009). Facile Synthesis of High‐Quality, Water‐Soluble, Near‐Infrared‐Emitting PbS Quantum Dots. European Journal of Inorganic Chemistry.

[22] Liang Y (2015). Capping nanoparticles with graphene quantum dots for enhanced thermoelectric performance. Chemical Science.

[23] Kim D, El-Shall H, Dennis D, Morey T (2005). Interaction of PLGA nanoparticles with human blood constituents. Colloids and Surfaces B: Biointerfaces.

[24] Yin J-J (2008). Inhibition of tumor growth by endohedral metallofullerenol nanoparticles optimized as reactive oxygen species scavenger. Molecular Pharmacology.

[25] Fard JK, Jafari S, Eghbal MA (2015). A review of molecular mechanisms involved in toxicity of nanoparticles. Advanced pharmaceutical bulletin.

[26] Lewinski N, Colvin V, Drezek R (2008). Cytotoxicity of nanoparticles. Small.

[27] Shin SW, Song IH, Um SH (2015). Role of physicochemical properties in nanoparticle toxicity. Nanomaterials.

[28] Derfus AM, Chan WC, Bhatia SN (2004). Probing the cytotoxicity of semiconductor quantum dots. Nano letters.

[29] Hoshino A, Hanada S, Yamamoto K (2011). Toxicity of nanocrystal quantum dots: the relevance of surface modifications. Archives of toxicology.

[30] Pelley, J. L., Daar, A. S. & Saner, M. A. State of academic knowledge on toxicity and biological fate of quantum dots. *Toxicological Sciences*, kfp188 (2009). 10.1093/toxsci/kfp188 PMC2777075 19684286

[31] Sohaebuddin SK, Thevenot PT, Baker D, Eaton JW, Tang L (2010). Nanomaterial cytotoxicity is composition, size, and cell type dependent. Particle and fibre toxicology.

[32] Winnik FM, Maysinger D (2012). Quantum dot cytotoxicity and ways to reduce it. Accounts of chemical research.

[33] Chen N (2012). The cytotoxicity of cadmium-based quantum dots. Biomaterials.

[34] Jaishankar M, Tseten T, Anbalagan N, Mathew BB, Beeregowda KN (2014). Toxicity, mechanism and health effects of some heavy metals. Interdisciplinary toxicology.

[35] Jan AT (2015). Heavy metals and human health: Mechanistic insight into toxicity and counter defense system of antioxidants. International journal of molecular sciences.

[36] Cho, W.-S. *et al*. Zeta potential and solubility to toxic ions as mechanisms of lung inflammation caused by metal/metal-oxide nanoparticles. *Toxicological Sciences*, kfs006 (2012). 10.1093/toxsci/kfs006 22240982

[37] Luna-Velasco A, Field JA, Cobo-Curiel A, Sierra-Alvarez R (2011). Inorganic nanoparticles enhance the production of reactive oxygen species (ROS) during the autoxidation of l-3, 4-dihydroxyphenylalanine (l-dopa). Chemosphere.

[38] Porres L (2006). Absolute measurements of photoluminescence quantum yields of solutions using an integrating sphere. Journal of Fluorescence.

[39] Treves A, Heidelberger E, Feldman M, Kaplan H (1979). *In vitro* sensitization of human lymphocytes against allogeneic cells by antigen-fed macrophages. Israel journal of medical sciences.

[40] Shaki F, Hosseini M-J, Ghazi-Khansari M, Pourahmad J (2012). Toxicity of depleted uranium on isolated rat kidney mitochondria. Biochimica et Biophysica Acta (BBA)-General Subjects.

[41] Zhao Y (2010). Vanadium compounds induced mitochondria permeability transition pore (PTP) opening related to oxidative stress. Journal of inorganic biochemistry.

[42] Hissin PJ, Hilf R (1976). A fluorometric method for determination of oxidized and reduced glutathione in tissues. Analytical biochemistry.

[43] Tavakoli MM (2015). Physicochemical properties of hybrid graphene–lead sulfide quantum dots prepared by supercritical ethanol. Journal of nanoparticle research.

[44] Son DI (2012). Charge separation and ultraviolet photovoltaic conversion of ZnO quantum dots conjugated with graphene nanoshells. Nano Research.

[45] Tavakoli MM, Aashuri H, Simchi A, Kalytchuk S, Fan Z (2015). Quasi core/shell lead sulfide/graphene quantum dots for bulk heterojunction solar cells. The Journal of Physical Chemistry C.

[46] Bhattacharyya A, Chattopadhyay R, Mitra S, Crowe SE (2014). Oxidative stress: an essential factor in the pathogenesis of gastrointestinal mucosal diseases. Physiological reviews.

[47] Premanathan M, Karthikeyan K, Jeyasubramanian K, Manivannan G (2011). Selective toxicity of ZnO nanoparticles toward Gram-positive bacteria and cancer cells by apoptosis through lipid peroxidation. Nanomedicine: Nanotechnology, Biology and Medicine.

[48] Wang J, Sun P, Bao Y, Liu J, An L (2011). Cytotoxicity of single-walled carbon nanotubes on PC12 cells. Toxicology in vitro.

[49] Manke, A., Wang, L. & Rojanasakul, Y. Mechanisms of nanoparticle-induced oxidative stress and toxicity. *BioMed research international* **2013** (2013). 10.1155/2013/942916 PMC3762079 24027766

[50] Morin V (2008). Cathepsin L inhibitor I blocks mitotic chromosomes decondensation during cleavage cell cycles of sea urchin embryos. Journal of cellular physiology.

