# Supplementary material for: Biochemical mechanisms of dose-dependent cytotoxicity and ROS-mediated apoptosis induced by lead sulfide/graphene oxide quantum dots for potential bioimaging applications
Source: Sci Rep. 2017 Oct 10;7:12896. doi: 10.1038/s41598-017-13396-y (PMC5635035; doi:10.1038/s41598-017-13396-y)
Supplement: Supplementary file 1 — Electronic Supplementary Information [file 41598_2017_13396_MOESM1_ESM.pdf]

Electronic Supplementary Information (ESI)

Biochemical Mechanisms of dose-dependent cytotoxicity and ROS-mediated apoptosis induced by lead sulfide/graphene oxide quantum dots for potential bioimaging applications

Mahdi Ayoubi[a†], Parvaneh Naserzadeh[b†], Mohammad Taghi Hashemi[a], Mohammad Reza Rostami[a], Elnaz Tamjid[c], Mohammad Mahdi Tavakoli[a], Abdolreza Simchi[a,d*]

[a]*Department of Materials Science and Engineering, Sharif University of Technology, P.O. Box 11365-11155, Tehran, Iran*

[b] *Department of Pharmacology and Toxicology, Faculty of Pharmacy, Shahid Beheshti University of Medical Sciences, P.O. Box 14155-6153, Tehran, Iran*

[c]*Department of Nanobiotechnology, Faculty of Biological Sciences, Tarbiat Modares University, P.O. Box 14115-175, Tehran, Iran*

[d]*Institute for Nanoscience and Nanotechnology, Sharif University of Technology, P.O. Box 11365-11155, Tehran, Iran*

*Corresponding author: A. Simchi; Tel: +98 (21) 6616 5226; Fax: 6616 0057; E-mail: simchi@sharif.edu

†These authors contributed equally in this work

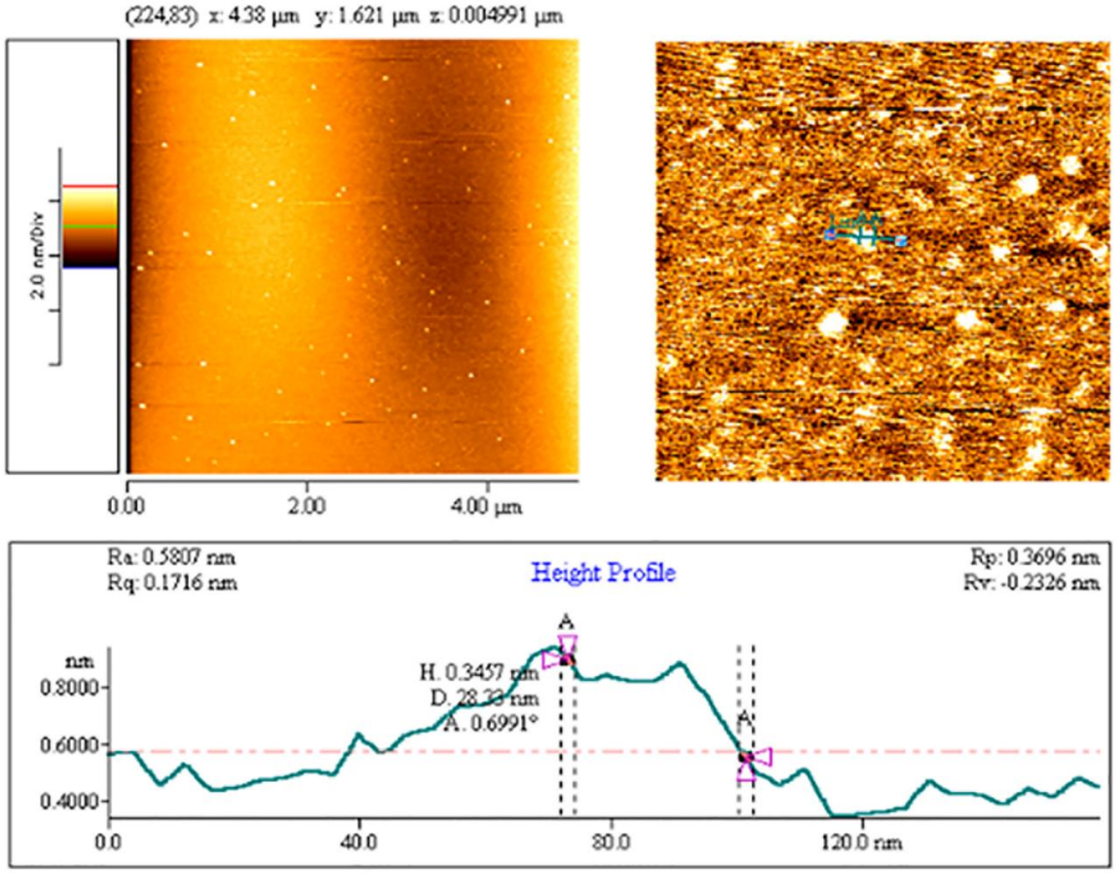

Figure S1: AFM images and height profile of graphene oxide dots prepared by electrolysis of the graphite rod in an aqueous solution of NaOH (0.1 M).

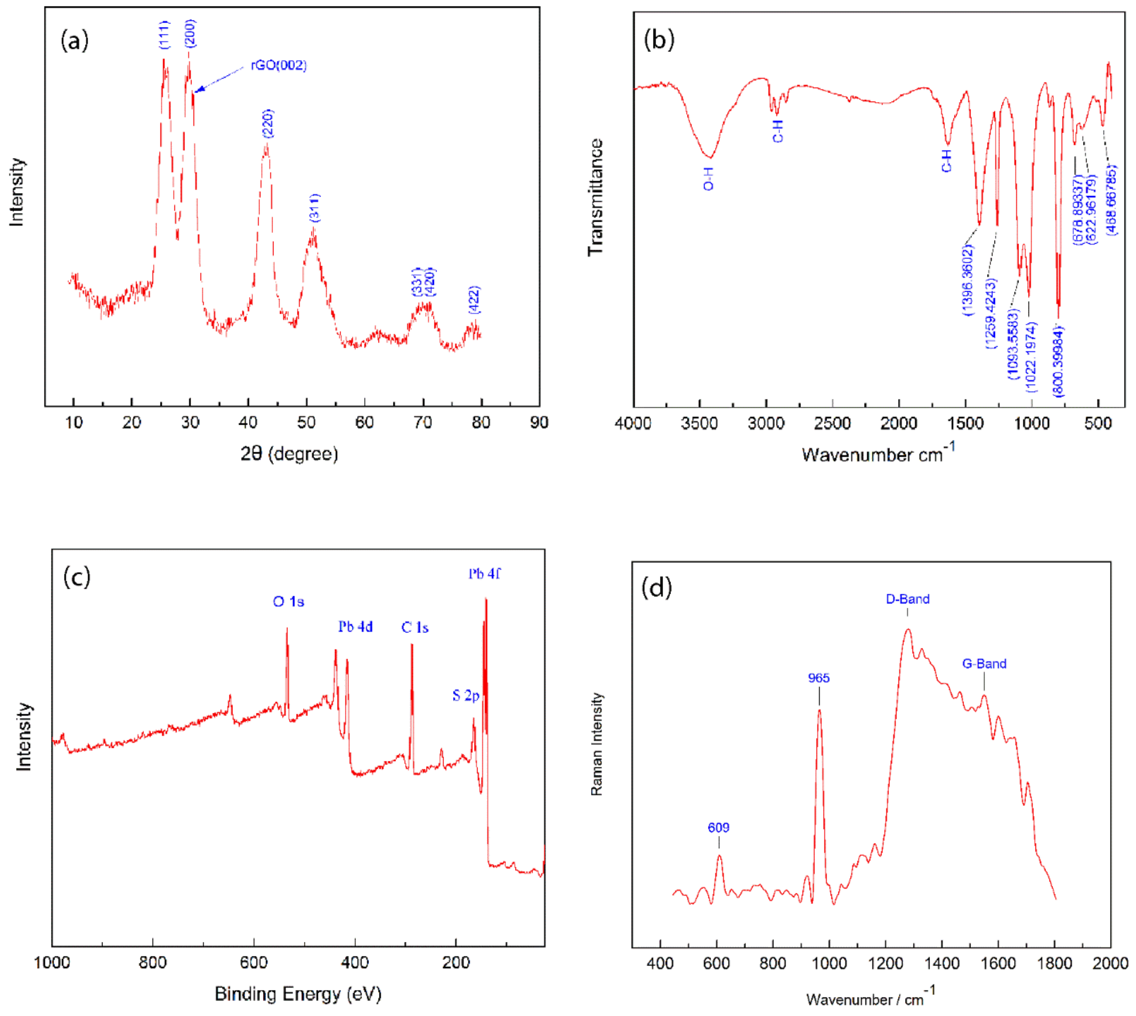

Figure S2: (a) XRD pattern, (b) FTIR and (c, d) XPS spectra of hybrid quantum dots.

Table S1. Fitting parameters used for TRPL measurement

Quantum dots	τ (μs)	β	χ
PbS	1.1	11.65	1.01
PbS/rGO	0.89	12.35	1.03
